# Computational Modeling and Reverse Engineering to Reveal Dominant Regulatory Interactions Controlling Osteochondral Differentiation: Potential for Regenerative Medicine

**DOI:** 10.3389/fbioe.2018.00165

**Published:** 2018-11-13

**Authors:** Raphaelle Lesage, Johan Kerkhofs, Liesbet Geris

**Affiliations:** ^1^Prometheus, Division of Skeletal Tissue Engineering Leuven, KU Leuven, Leuven, Belgium; ^2^Biomechanics Section, KU Leuven, Leuven, Belgium; ^3^Biomechanics Research Unit, GIGA in silico Medicine, University of Liège, Liège, Belgium

**Keywords:** *in silico* modeling, gene regulatory network, network inference, chondrocyte, differentiation, regenerative medicine

## Abstract

The specialization of cartilage cells, or chondrogenic differentiation, is an intricate and meticulously regulated process that plays a vital role in both bone formation and cartilage regeneration. Understanding the molecular regulation of this process might help to identify key regulatory factors that can serve as potential therapeutic targets, or that might improve the development of qualitative and robust skeletal tissue engineering approaches. However, each gene involved in this process is influenced by a myriad of feedback mechanisms that keep its expression in a desirable range, making the prediction of what will happen if one of these genes defaults or is targeted with drugs, challenging. Computer modeling provides a tool to simulate this intricate interplay from a network perspective. This paper aims to give an overview of the current methodologies employed to analyze cell differentiation in the context of skeletal tissue engineering in general and osteochondral differentiation in particular. In network modeling, a network can either be derived from mechanisms and pathways that have been reported in the literature (knowledge-based approach) or it can be inferred directly from the data (data-driven approach). Combinatory approaches allow further optimization of the network. Once a network is established, several modeling technologies are available to interpret dynamically the relationships that have been put forward in the network graph (implication of the activation or inhibition of certain pathways on the evolution of the system over time) and to simulate the possible outcomes of the established network such as a given cell state. This review provides for each of the aforementioned steps (building, optimizing, and modeling the network) a brief theoretical perspective, followed by a concise overview of published works, focusing solely on applications related to cell fate decisions, cartilage differentiation and growth plate biology. Particular attention is paid to an in-house developed example of gene regulatory network modeling of growth plate chondrocyte differentiation as all the aforementioned steps can be illustrated. In summary, this paper discusses and explores a series of tools that form a first step toward a rigorous and systems-level modeling of osteochondral differentiation in the context of regenerative medicine.

## Introduction

In the growing world of regenerative medicine, the ability to robustly control cell differentiation processes becomes increasingly important. This also applies to regeneration of osteochondral tissues since controlling cell fate decision and differentiation of chondrocytes might have great benefits for both bone defects and cartilage degenerative diseases.

Most of the mammalian skeleton is composed of bone that is formed through endochondral bone formation starting from a cartilaginous template. During development, recruited mesenchymal stem cells undergo condensation. Then, with the influence of a number of factors, the cells start to differentiate into chondrocytes and secrete cartilage matrix rich in type II, IX, and XI collagen. This stage in the chondrogenic differentiation cascade is marked by the expression of the transcription factor SOX9 (Lefrebvre and de Crombrugghe, 1998; Hata et al., 2017). The chondrogenic cells continue to proliferate in a columnar structure and at a certain stage they exit the cell cycle to undergo hypertrophy at the center of the condensation (primary ossification center). This event is associated with secretion of type X collagen (O'Keefe et al., 1994), mineralization of the extracellular matrix (ECM) and expression of molecular markers such as the transcription factor RUNX2, the matrix metalloproteinase MMP13 and the vascular endothelial growth factor VEGF. Although it is commonly accepted that hypertrophic cells tend to undergo apoptosis and be replaced by osteoblasts (bone forming cells), it is now confirmed that a certain percentage transdifferentiates into osteoblasts (Yang et al., 2014). The ensuing vascular invasion, degradation of the mineralized matrix and the production of bone matrix together achieve the bone formation. The same processes also occur at the articular end of the bone (the secondary ossification center), such that a zone of chondrocytes persists only between the primary and secondary ossification centers, called the growth plate. The growth plate has a columnar organization with zones of proliferating chondrocytes, hypertrophic chondrocytes, and bone formation (Long and Ornitz, 2013).

At the adult stage, the only hyaline (stable) cartilage found in long bones, in skeletal homeostasis, is at the joint surface. These chondrocytes do not undergo hypertrophy but remain in a stable phenotype characterized by a low rate of proliferation and the production of ECM rich in Col-II and Aggrecan. However, some degenerative diseases such as osteoarthritis have been associated with dysregulation of the stable cartilage where both the chondrocyte's rate of proliferation and its switch toward hypertrophy are modified leading to abnormal ossification of the joints.

A variety of possible treatment strategies are currently under investigation, both curative and restorative, with amongst them the use of drugs to inhibit the abnormal switch toward hypertrophy or the use of cartilage-engineered constructs to replace affected osteochondral tissues and promote regeneration. Indeed, tissue-engineered (TE) constructs are being developed to treat large tissue defects where spontaneous healing has failed.

Lenas et al. (2009a) presented the paradigm of “developmental engineering” arguing that a better understanding of the developmental skeletal tissue formation process and a better control of the developmentally-inspired *in vitro* process of chondrogenic differentiation will help to develop more qualitative and robust bone and cartilage TE constructs. In both aforementioned treatment strategies (drugs and TE), the ability to precisely control the cell differentiation and the switch from one genetic program (SOX9) to another (RUNX2) is implied.

Computer models provide a formal framework to study the dynamics of genetic programs within a cell. Computational biology is the field where informatics, engineering and biology meet to enhance the understanding of biological systems and, notably, their underlying regulatory networks (RN). Computer modeling can be used to interpret experimental findings, to help in the design of new experiments and to identify potential therapeutic targets. The importance of systems biology in the field of tissue engineering and regenerative medicine has increased over the last years (e.g., Sengers et al., 2008; Lenas et al., 2009b; Geris et al., 2010; Rajagopalan et al., 2013; Carlier et al., 2014; Geris, 2014; McNamara et al., 2015; Smeets, 2016). A variety of modeling technologies is being used, covering processes at different spatial and temporal scales (genes/protein, cell, tissue, organs, systems, …). One family of models, the (gene) regulatory network (G)RN models, may be of particular interest when it comes to deciphering signaling and the cell response implied in cell fate decision. Indeed, the human intuition is limited in its capacity to deal with the complex interplay present in signaling networks whereas a growing arsenal of *in silico* models shows how formal computer language can help to tackle these issues. The works of Aldridge et al. (2009), Saez-Rodriguez et al. (2009), Woolf et al. (2005), or Xia et al. (2006) are a non-exhaustive list of examples where *in silico* models were successfully used to unravel biological complexity and give new biological insights.

**Figure 1:**
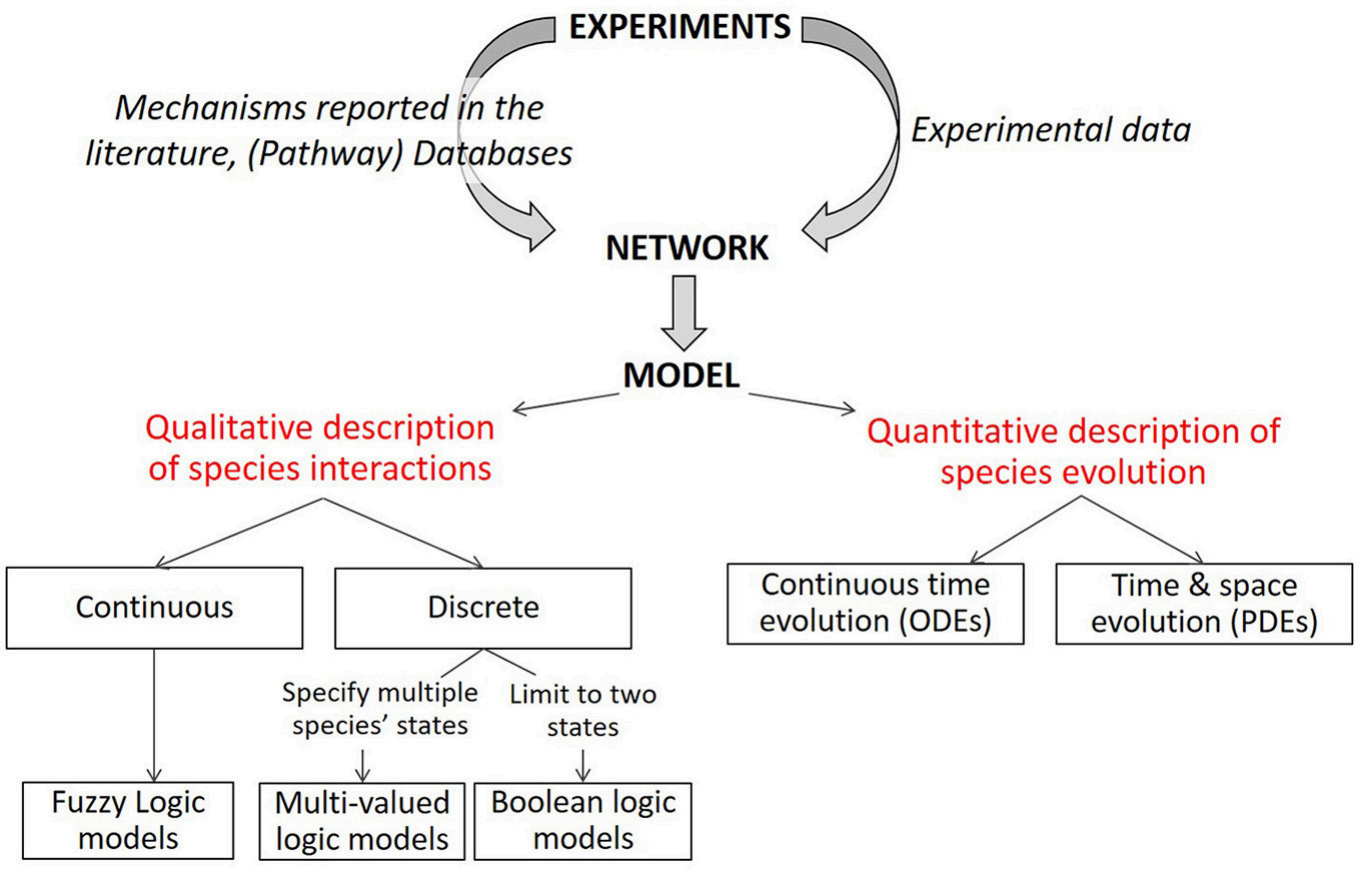
Description of modeling formalisms. Starting from a static network graph obtained from experimental data, various modeling approaches can be used to simulate the evolution over time of the network components. Quantitative models describe the evolution of species over time with ordinary differential equations (ODE) and can introduce spatial resolution with partial differential equations. Qualitative models (limited here to logical models) describe the evolution of species in terms of logical statements. Discrete logic can specify two or more levels for each modeled species (only two for Boolean logic). Various methods of describing discrete or Boolean logical models with piece-wise continuous equations or logic-based ODEs have been successfully implemented to represent biochemical signaling networks. Modified from Morris et al. (2010).

In this review we aim to provide an overview of the different methods that can be employed to generate such (G)RN models (see overview Figure 1). The first step is the generation of a network graph, which can be either derived from mechanisms and pathways that have been reported in the literature (knowledge-based approach) or it can be inferred directly from the data (data-driven approach). Combinatory approaches allow further optimization of the network. This network graph provides a static (unchanging) picture of the biological process under study. Once a network is established, several modeling technologies are available to interpret dynamically the relationships that have been put forward in the network graph. A dynamic analysis amounts to simulating the evolution over time of the different network elements under specific conditions and to studying the possible outcomes (stable states) of the established network. We will start this review with the modeling part and subsequently discuss the network part. In each case, we will start by presenting the general principles and some of the computational methods currently available, followed by a non-exhaustive overview of the studies that already made use of those computational modeling approaches to study cell fate decision, mostly focusing on applications of chondrocyte differentiation and growth plate dynamics. Finally, we will discuss different strategies where knowledge-derived modeling approaches and data-based approaches can be used together to complement each other. This combination strategy will be illustrated by a case study focusing on a model of chondrocyte differentiation in the growth plate.

Here, we focus on regulatory networks at the intracellular level where e.g., the ECM and mechanical forces are represented by the intracellular signals they generate which activate the corresponding pathways in the regulatory network. Providing an overview of existing models at other spatio-temporal scales that can take these factors (ECM, mechanics etc.) into account in a more explicit manner, is beyond the scope of this paper. We refer the readers to review papers on the subject of multiscale modeling of skeletal tissue engineering and regeneration processes (Glimm et al., 2012; Julkunen et al., 2013; Geris, 2014; Yousefi et al., 2015). This review ultimately should provide biologists with the necessary vocabulary and information to understand the requirements and accomplishment of (G)RN models.

## *In silico* knowledge-based modeling of regulatory networks to study cell fate decision

Mechanistic network-based models start from a static network and use knowledge and mechanisms gleaned from decades of biomedical experimental research to bring this static network toward a dynamic mathematical model. A mathematical model describes a system, for instance a biological system, using mathematical concepts. A model is composed of a set of variables and a set of equations. The variables are quantities with a value that can change according to the equations that establish rules between the variables. The equations are built using an ensemble of parameters with a given (fixed) value; they determine how variables evolve in simulations. It is not easy to determine the most appropriate *in silico* technology amongst all of the available *in silico* modeling methods; which is why several teams tackled this issue by proposing different kinds of classifications. Some of these classifications are based on the mathematical methodology, others are based on biological issues. Janes and Lauffenburger (2006) proposed a decision tree to classify and help to choose the methods best suited for answering a particular question according to various criteria. After a general introduction, the following sections will present some of these methods by classifying them into commonly accepted mathematical subgroups. This classification into subgroups is summarized in the lower part of Figure 1, adapted from Morris et al. (2010), showing the different mechanistic modeling approaches which are described in the text below starting with the distinction between quantitative and qualitative models.

### General principles and formalism of knowledge-based modeling

#### Quantitative models (differential equations)

Usually biologists build regulatory pathway maps as a static representation of the knowledge they get from experiments while the goal of systems biology is to turn them into dynamic models. It means that the model should be “executable” through simulation instead of being a simple map. A common way to study a system's behavior in a quantitative way is by using differential equations (Box 1). In systems biology, the ordinary differential equations (ODE) describe changes over time whereas the partial differential equations (PDE) describe changes in space and time (Wolkenhauer et al., 2005). In order to capture the concentration changes of molecules over time and describe the interaction between variables, the differential equations rely on the law of mass action and its derivations such as the Hill equation. For RN, these mathematical formulations state that the expression level of a gene at time *t*+*1* depends on the weighted expression levels of other genes at time *t*, making them perfectly suited to represent changes in levels of gene expression over time. The same formalism can be used to simulate changes in protein activity or protein concentration levels over time due to interactions with other proteins. The mathematical equations, describing these evolutions over time, contain parameters that are related to the network topology (i.e., the way the biological components are connected to each other) and to the strength of regulation (Liu et al., 2012). For instance when time-varying ODEs are used to represent biochemical reactions and a network of protein interactions, parameters typically are dissociation constants, kinetic rate constants, reaction order, etc. It is possible to add further terms/parameters that indicate the influence of additional substances (Schlitt and Brazma, 2007) if the purpose is, for instance, to simulate drug therapies. For a formal description of how to use ODEs in order to build a GRN model (see Klipp et al., 2005; Wolkenhauer et al., 2005).

Box 1 **Definitions of modeling types—in the context of systems biology applications**

**Deterministic:** A deterministic model is a model that will give the same output each time it is run with the same starting conditions.

**Stochastic:** By opposition to deterministic, in a stochastic model, one and the same initial state can lead to many different trajectories. This randomness is often used to represent biological uncertainty.

**Differential equation:** an equation relating a variable and its derivative(s).

**Ordinary differential equation:** In systems biology an ODE most commonly describes the evolution of a variable over time in function of other variables.

**Partial differential equation:** PDEs in systems biology typically describe the evolution of a variable with respect to time and space.

**Multifactorial data:** In GRN context, this is data obtained by slightly perturbing all genes simultaneously. It can typically be expression profiles coming from different patients or biological replicates. Multifactorial data are the most common data source as they are easier to obtain than knockout or time series-data.

A simple way to introduce a spatial component when time-varying ODE equations are used, is through compartmentalization. The same variables occurring at different locations within the cell are simulated as distinct variables. For instance, in kinetic models, the same proteins present in different cell compartments (organelles) are simulated as distinct variables and membrane diffusion or speed of translocation are the additional parameters associated with the compartmentalization (Janes and Lauffenburger, 2006). This is interesting, for instance, in the case of proteomic models where molecules can translocate from one organelle to another. When translocation occurs, there could be an import/export mechanism generating a time delay that could have a significant influence on the behavior of the system, which can be captured by appropriate mathematical formulations (Wolkenhauer et al., 2005).

Important to remark is that ODE models depend on numerical parameters and initial conditions that are often difficult to measure (Wolkenhauer et al., 2005). That is why modelers usually perform sensitivity analyses. It means that one tries to assess how much a result can change when varying one or several of the parameters. For robust systems, the exact value of a particular parameter may not be essential. Robustness with respect to parameter values seems to be a typical property of most models capturing biological behavior, also termed “sloppiness” as explained in Gutenkunst et al. (2007). Studying the robustness of the system allows determining for which parameters an accurate value is necessary to obtain reliable model predictions and for which parameters that is not the case.

When there is a discrepancy between the experimental observations and the simulation results from models built on curated knowledge such as published protein interactions, the model can be used to suggest possible unknown interactions between the model variables that could help to better explain the experimental data. In that case, the hypothetic interactions proposed can be tested experimentally, which constitutes the iterative process that governs systems biology (McNamara et al., 2015). Following this approach, differential equation based models have been proven able to lead to new testable hypotheses. For instance, von Dassow et al. (2000) developed a differential equation system of developmental processes in Drosophila including 48 parameters (half-lives of messenger RNAs and proteins, binding ranges, etc.). The initial model described all known interactions, but the addition of at least two new hypothetical interactions was needed to ensure the results were fitting the experimental observations reported in the literature.

#### Qualitative models

Qualitative models do not aim to provide exact values of concentrations of network components. They rather aim to capture the overall qualitative behavior of networks. In logical models, variable values are the result of logical relationships (AND, OR, NOT gates) with other variables. Logical modeling was first applied to model GRNs by Stuart Kauffman (Kauffman, 1969, 1994; Glass and Kauffman, 1973). In Kauffman's approach, variables are evaluated using logical combinations of other variables and each variable can take only a discrete number of values (0 or 1 for Boolean models). René Thomas, another pioneer in logical models in systems biology, further refined the logical formalism by introducing multivalued variables and logical parameters equivalent to the kinetic parameters from differential equations (Thomas and Kaufman, 2001). This enabled him to introduce asynchronous updating of the system, which was later extended using temporal logic (Bernot et al., 2004). Indeed, logical systems can be updated in a synchronous way where all variables are updated at the same time during each transition, or in an asynchronous way where values of variables are updated one after the other. The ensemble of successive states through which a system passes during a simulation is called a trajectory and it can vary according to the initial state and the chosen updating strategy. Tracing all the trajectories across the ensemble of states allows to build a state-transition graph (Le Novère, 2015). With logical models, systems biologists usually study the stable states of the system, meaning that they try to identify in which stable state the system tends to settle. A stable state can be a fixed point or a discrete number of states in between which the system oscillates (e.g., for circadian rhythms the baseline behavior is a cyclic one). A system can settle in different stable states according to the initial condition from where it leaves. The ensemble of initial states leading the system to settle in the same stable state, is called the basin of attraction of the corresponding stable state (Mojtahedi et al., 2016). In regulatory networks theory, a system is characterized by its specific stable states. The study of the nature of the stable states and of their basin of attraction can give insight about cell states and cell state reachability, with application in cell fate decision and differentiation. Indeed, the size of a basin of attraction, meaning the number of initial states leading to a stable state, may give an estimate of how likely it is for a cell to reach this state (Abou-Jaoudé et al., 2016). For explicit cases of application see sections Current Computational Models as Predictive Tools for Cell Differentiation and Intracellular Regulatory Network Models of Growth Plate Cells.

Upstream regulators of one specific biological entity (gene/protein) and the ways they regulate it, may vary between different possibilities over time, depending on, for instance, external inputs and the concentrations of the regulators themselves. This behavior cannot be captured by the standard Boolean networks described in the previous paragraph. Probabilistic Boolean networks have been introduced in order to capture this uncertainty in the regulatory logic. In practice, the same initial state can lead to many different trajectories due to the stochastic nature of the model (Box 1). This is achieved by introducing several possible mathematical regulation functions (logical combinations of other biological entities) with different probabilities for each entity. At each time step, an entity is updated following one of its different regulation functions chosen randomly (Karlebach and Shamir, 2008). Another probabilistic approach is the Bayesian network but the dynamic aspects are not considered in these models since they often take the form of a directed acyclic graph (Liu et al., 2012). Karlebach and Shamir (2008) proposed dynamic Bayesian networks as a way to counter the lack of dynamic resolution of regular Bayesian networks.

Another good way to analyze model dynamics is through the use of Petri Nets since in that representation, nodes are not biological entities but places (= conditions) or transitions (= events) while directed arcs connect input places to transitions and transitions to output places. The number of transitions to reach a specific state can hence be assessed easily. This approach shows good results of prediction for some systems as illustrated in Steggles et al. (2007). It has the advantage of having an easy graphical representation, which makes this formalism a good common ground for biologists and mathematicians/modelers alike.

Whereas, differential equation based models of GRNs and signaling pathways suffer from the lack of kinetic information (Le Novère, 2015), qualitative models require a smaller amount of data (Karlebach and Shamir, 2008) and qualitative experimental observations might even be sufficient. Qualitative models constitute a good starting point when some interactions of the network remain unknown and it is easy to analyze variants of the same network (Ay and Arnosti, 2011). Indeed, despite the growing amount of biological knowledge about various biological phenomenon, it is not rare that the exact mechanisms remain not fully understood or even controversial. With missing detailed information it is often difficult to develop a mechanistic quantitative model. However, the missing information might not be detrimental to build a qualitative model which catches the wanted behavior, or, alternatively, allows to test different biological scenarios for their potential to capture the wanted behavior. Moreover, once a model explains an actual cell behavior, it becomes possible to study this system under various stress or perturbed conditions to give new predictions.

#### Fuzzy logic and alternative classifications

Certain model approaches are situated between the previously discussed categories of quantitative and qualitative. Fuzzy logic is one of those model approaches that technically might be considered to be qualitative but allows to include more complexity, making it closer to semi-quantitative approaches. When the behavior that needs to be captured is too complex to capture with discrete logical models, fuzzy logic models allow to introduce continuous regulation as well as provide the capacity to handle a “graded truth.” While Boolean logic takes into account only the values of 0 or 1 to describe the variables state (or additional discrete values in a multi-value discrete model), fuzzy logic accepts any value between 0 and 1. The main idea underlying fuzzy logic is that both subjective/abstract and objective knowledge can be integrated to solve a problem (Mendel, 1995). Indeed, the fuzzy logic can integrate intermediate values and even words, such as “low” and “high” concentrations (subjective knowledge) as suggested by Zadeh (1996). This is of particular relevance to the modeling of biological networks since in this field, the information is sometimes subjective and imprecise, and it becomes difficult to use clear mathematical or logical values to express it. Additionally, it enables very naturally to model uncertainty in signaling networks. Aldridge et al. (2009) provide a good example of adaptation of fuzzy logic to cellular signaling network analysis. In that study the authors investigate the relationship between 2 signaling pathways that may account for the previously known influence of the protein MK2 in cancer cell survival whilst the exact mechanism was not yet fully deciphered. The fuzzy logic approach enabled them to incorporate qualitative (abstract) data drawn from literature such as “low,” “medium,” “high” state of variables and still produce quantitative predictions. They even used a time variable influencing the output state of some proteins with a “low” value for time referring to early signaling responses (0–2 h) and a “high” value referring to late signaling events (2–24 h).

### Application to cell fate decision and osteochondral differentiation

The various formalisms described above have been applied frequently for prediction of pluripotent and stem cell fate decision with application in regenerative medicine (Pir and Le Novère, 2016). This section gives an overview of different published models, focusing on (stem) cell fate decision in general and on chondrocyte differentiation and growth plate dynamics in particular.

#### Current computational models as predictive tools for cell differentiation

The model of Schittler et al. (2010) is a good example of a quantitative model predicting osteochondral cell fate decision. Indeed, they use a GRN, mathematically implemented with differential equations, for both single cell scenarios and cell population scenarios to investigate an osteochondral differentiation system. They modeled the switch mechanisms between three stable states, being the progenitor, osteogenic and chondrogenic states.

Since activation and loss of specific genetic programs governs cell fate decisions, logical GRN models may also be a tool of choice for studying and predicting cell differentiation. Indeed, in this formalism, nodes or variables represent genes or the activity of transcription factors (TF), and connections between the nodes represent regulatory interactions (activation or inhibition) between them. During simulation the model can reach some stable states (cf. section Qualitative Models), which can be considered to equate to a specific (mature) cell types (Glass and Kauffman, 1973). This corroboration between stable state of the network and cell phenotype is the major hypothesis at the basis of all possible cases of application of regulatory network studies.

Herberg and Roeder (2015) reviewed qualitative (Boolean) GRN modeling methods to study embryonic stem cells differentiation where the analysis of the landscape of states and transitions between states gave great insight into the dynamics governing cell fate decisions. The usefulness of random Boolean networks in representing cell type convergence was even extended by Bodaker et al. (2013) with a study showing tissue-like regeneration in multi-cellular organisms. Indeed, in that paper, each cell constituting the multi-cellular organism was represented by a random Boolean network at the intracellular level and the study was based on the fact that the function of cells, and so their differentiation, can be altered following the influence of external signals from a neighboring population of cells on their individual intracellular networks. This approach opens perspectives on how to integrate the influence of neighboring cells and extracellular signals in Boolean networks, which are typically used to study cells in isolation. Other examples of network models enabling predictive analysis of mesenchymal stem cell differentiation into chondrocytes and osteoblasts under various biochemical conditions can be found in Woolf et al. (2005), who used Bayesian networks as modeling technology.

#### Intracellular regulatory network models of growth plate cells

**Figure 2:**
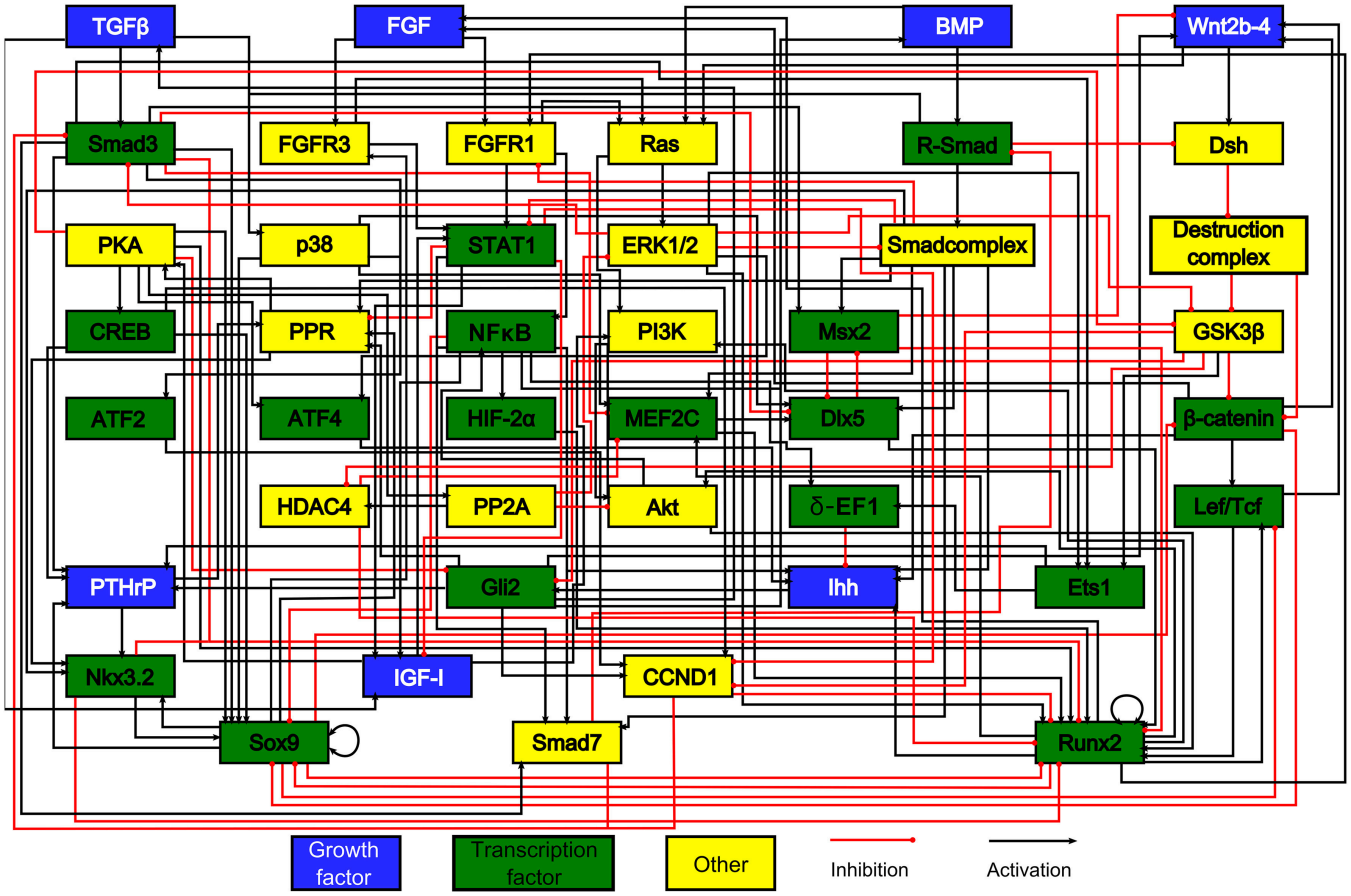
The model's chondrocyte gene network. Every box represents a gene, its protein or in some cases a complex of them. The interactions are represented by red and black lines if they are inhibitory and stimulatory, respectively. Blue boxes denote growth factors, green boxes are transcription factors, yellow boxes do not belong to either category. Reproduced from Kerkhofs and Geris (2015).

To date, very few *in silico* skeletal models have focused on the growth plate—especially at the intracellular level. Kerkhofs and coworkers implemented a series of models on the genetic switch between the chondrocyte's proliferative and hypertrophic state within the growth plate (Kerkhofs et al., 2012, 2016; Kerkhofs, 2015; Kerkhofs and Geris, 2015), The control of this switch was studied both in the context of regenerative medicine and tissue engineering, and in the context of degenerative cartilage diseases (Melas et al., 2014). The model that we originally developed (Kerkhofs et al., 2012) was an additive, multi-valued, Boolean model representing the genetic switch from a SOX9 positive stable state to a RUNX2 positive stable state, being the hallmark of the proliferative and the hypertrophic state of the chondrocyte, respectively (see section Qualitative Models for formal definition of stable states). Given that lack of human data, the network used in the Kerkhofs models was built mostly using mouse data (Figure 2). This network model gave a first *in silico* insight into the genetic regulation underlying chondrocyte phenotypes within the growth plate and was successful in capturing the effects of knockouts in the main regulatory pathways of the growth plate regulation described in the literature. In subsequent studies, the model was improved by adding (1) a quantitative resolution and (2) temporal priority classes that are absent in classic Boolean models. Adding quantitative resolution, meaning that each node can have any value ranging between 0 and 1, was handled through the implementation of an additive framework. In an additive approach, the value of each variable (= protein activity or gene expression level) is updated by a weighted sum of the values of upstream variables. The temporal resolution of the reactions was managed by incorporating priority classes to account for different reaction kinetics. All reactions related to slow processes such as mRNA or protein production, were referred to as slow reactions (lower priority) and those related to fast processes such as protein activity, were referred to as fast reactions (higher priority). For each node of the network, a fast (“protein activity”) and slow (“gene expression”) variable was defined with the total activity of any given node being the product of the fast and slow variable. When performing the model (asynchronous) updating, high priority interactions are taken into account first, before taking into account the lower priority interactions, reflecting the time difference between these categories of processes in cellular systems. This approach enabled to capture dynamics and behaviors more complex than those found with classic Boolean models. For instance, in Kerkhofs and Geris (2015) we showed that the model was able to simulate dose response studies where different levels of stimuli in proliferative chondrocytes gave rise to qualitatively different responses. The framework was used furthermore to perform an analysis of the stable states and a stable state perturbation analysis, assessing the influence of specific factors of the network thanks to *in silico* over-activation or KO. We also investigated the relevance of the modeling results in osteoarthritis as abnormal chondrocyte hypertrophy plays a role in this disease (Kerkhofs et al., 2016). The perturbation study may point out potential key biological factors to be targeted experimentally to either promote the differentiation or to inhibit it.

The Kerkhofs models were implemented in a general computational software package called MATLAB (The Mathworks). In order to make the models easily accessible to the wider audience of non-modeling specialists, the interface is of great importance. Scholma et al. (2014) and Schivo et al. (2016) implemented the Kerkhofs models into a timed automata framework, called ANIMO. This modeling framework is available as a plug-in of Cytoscape and has been conceived particularly around its intuitive end user interface to facilitate use by a wider audience, including biologists.

## Data driven modeling-network inference

The preceding section described methods used to generate model predictions, once a network has been established. This section will focus on establishing such networks. There are two main classes of approaches. In one approach (knowledge-based, literature-curated), networks are built using mechanisms described in the literature and aggregated in (pathway) databases such as the network used in the Kerkhofs models described above. In the other approach (data-driven), networks are inferred directly from experimental data. The difficulty with the latter approach is to find/perform experiments with a sufficiently high information-content, typically requiring a standard condition with sufficient and sufficiently strong perturbations of that standard condition. This section will focus on the inference process and its challenges.

### General principles of network inference

#### The inverse problem and adaptation to network inference

In inverse problems, one aims to infer the parameters describing the system, given actual observations. In order to solve an inverse problem different methods can be used, depending on the exact nature of the question to be answered. Generally, inverse problems deal with an optimization problem since one needs to minimize a functional error between real values (data) and the simulated values (Tarantola, 2006; Liu et al., 2012).

Network inference can be considered as an inverse problem where the network parameters, being the absence, presence and direction of regulatory interactions, are derived directly from experimental data (Villaverde and Banga, 2013). With the huge progresses in the field of molecular biology these past few years, we currently dispose of large amounts of quantitative data such as mRNA levels, protein levels, phosphorylation states, etc. to use for model inference. However, it also raises the question of the identifiability of the network's parameters i.e., the ability to find an unambiguous set of parameters determined by the available data set, as, in many cases, several sets of parameters might fit the same data set.

#### Concise overview of methods for network inference

The development of a myriad of methods, formalisms and software tools helps to tackle the problem of inferring a network from microarray expression data as well as from RNA Sequencing data, protein-DNA binding data (ChIP-seq), CpG methylation, promoter sequence detection and proteomics data. Here we review a number of these methods following the categorization in four parts as found in Marbach et al. (2012) and Le Novère (2015). These categories are (1) the statistical methods, (2) probabilistic methods, (3) information theoretic methods, and (4) methods based on ODEs. Most of them propose, as an output, a ranked list of regulatory interactions from the one most likely to be a true interaction to the least likely one. Those interactions can represent protein interactions or associations, in the case of signaling network, but also interactions between transcription factors (TF) and genes in the case of gene networks. These interactions might be direct or indirect relationships. Details on the mathematics of the different methods for gene network inference can be found in Supplemental Data 1.

Most of these methods can work with multifactorial data (Box 1) and data of different types such as steady state and/or time series data. This is useful when considering the range of available data that often comes from experiments of one or multiple factor perturbations. Hence, combining different inference methods together (hybrid methods) might help to widen the scope of the type of data that can be taken into account. Moreover, the rationale behind GRN inference is to decipher the underlying existing regulatory network solely from perturbed gene expression data. Therefore, it is important to make sure that the biological material used to generate the data actually reliably reflects the biological system under investigation. This means that single-cell RNA-seq data is possibly the best way to ensure that the expression data does not capture the behavior of several heterogeneous systems but only that of the system of interest (Griffiths et al., 2018). For chondrocyte in the growth plate, the use of single-cell RNA-seq allowed the identification of regulatory molecular cascades important for the different stages of chondrocytes during development (Li et al., 2016). Nevertheless, single-cell data is not always available and inference from micro-array data or general RNA-seq data has been proven to perform well.

#### Strengths and weaknesses of inference methods and the ensemble approach

The amount of inference methods has been increasing in recent years (Marbach et al., 2009). De Smet and Marchal (2010) argue that direct integration of inference tools in daily laboratory practice by biologists is still limited, because the choice of the inference is not always obvious. Hence, the authors propose a new classification of the inference tools based on their nature, i.e., supervised/unsupervised, integrative or not, direct inference or based or pre-module construction etc., which gives new insights in the strength and weakness of each methods. Several studies tend to highlight the advantages and weaknesses of various methods for specific applications, but some studies aim to compare multiple methods. Since 2006, a big consortium called “the Dialogue on Reverse-Engineering Assessment and Methods (DREAM) challenge” tries to develop an objective assessment of reverse engineering methods for biological networks (Stolovitzky et al., 2007; Saez-Rodriguez et al., 2016). During the yearly DREAM challenges, scientists of the field from all around the world are invited to use the inference method of their choice to a given data set following specific guidelines. In that way, the organizers can apply blinded cross-comparisons of the results with standard metrics and they are able to identify the specific strengths and weakness of each method. Such assessment will help systems biologists to choose the inference method best suited to the question they aim to answer, given the type of data they have at their disposal. Additionally, it provides teams trying to develop new inference methods with a more formal assessment process, a new way to test their own algorithm (Stolovitzky et al., 2007).

In later DREAM Challenges (Marbach et al., 2012), revealed that no single inference method performs optimally across diverse datasets but predictions from multiple inference methods combined do. Indeed, they have estimated the performance of a consensus network constructed as an average network of multiple inference methods and they showed it was performing better than other methods taken individually. The consensus network is then the result of a voting approach in which the top ranked interactions predicted by all methods, meaning the most likely to exist according to all methods, are maintained in a new network. In combining predictions made by disparate methods, the intersection of their predictions complements their strengths and their weaknesses. Voting was shown to be an effective strategy for network inference, but it should be said that rather than finding the “best” network, it serves to find the “least bad” one (Marbach et al., 2012; Kerkhofs, 2015).

Finally, inference algorithms offer a systematic way to infer regulatory networks directly from data without the need to curate literature manually, a process that inadvertently introduces curator-bias. Increasing performance of the published algorithms, improvement in the field of machine learning and consensus approaches gathering several methods, together enhance the reliability, and accuracy of such inferred regulatory networks. This enables to produce a static map which can further be used as a starting point for computational simulations thanks to the modeling approaches described in section 2. Indeed, *in silico* simulations of such regulatory networks might give meaningful insight in the understanding of biological processes. Molinelli et al. (2013) provide a good example of a regulatory network inference process giving meaningful biological insight in the field of cancer. In their study, the authors first perform a large screening of experimental perturbations for high throughput measurement of proteomic changes (e.g., reverse phase protein arrays or mass spectrometry) and phenotypic changes (e.g., cell viability or apoptosis). They use this data as input to infer a network model of signaling in a RAF inhibitor resistant melanoma cell line (SKMEL-133). The constructed network enabled the authors to retrieve known pathways such as PI3K/Akt or MAPK pathways as well as new interactions that are consistent with known protein functions. Finally, from the network, they perform simulations of different *in silico* single or pairwise perturbations of nodes that were experimentally targeted or not in the input data. Their model is predictive of both the proteomic and phenotypic responses to drug combinations. Additionally, it successfully predicts the phenotypic response profiles of SKMEL-133 cells to novel drug targets, for instance, *in silico* simulations predicted that PLK1 inhibition was decreasing cell viability, which was validated *in vitro* with 99% of the cells eliminated with a 15 nM concentration of PLK1 inhibitor. Overall, this study uses the typical pipeline of *in vitro/in silico* integration to obtain new biological insights. Similar methodology could be of great interest to be applied to cell fate decision and osteochondral differentiation in order to identify the underlying regulatory networks involved and the key factors to target in order to modulate those processes. Therefore, some examples of network inference in this context are developed in the next sections.

### Application to cell fate decision and osteochondral differentiation

#### Inference of a growth plate network following a consensus approach

As a first illustration of inference methods and the consensus approach for osteochondral system, we return to the growth plate model described in section Intracellular Regulatory Network Models of Growth Plate Cells. We describe the inference process that we have followed, in a previous study, to build a data-driven network in order to validate the literature-curated one (Kerkhofs, 2015). The inference was executed using measurements from the mouse growth plate exclusively, as the topology of the literature-derived network from Kerkhofs et al. (2016) was derived mostly from studies performed in mice. An overview of the micro-array data can be found in Table 1. As can be seen, not all studies have divided the growth plate in the same zones, but the proposed network should be valid throughout the entirety of the growth plate. All the published experimental data were generated with Affymetrix Mouse 430.2 microarrays. All samples were normalized using the Guanine Cytosine Robust Multi-Array Analysis (Wu and Irizarry, 2004; Wu et al., 2004).

**Table 1:** Summary of literature sources for microarray data on the growth plate.

**Origin**	**Samples**	**Study design**	**Reference**
Primary growth plate chondrocytes	12	Cells treated with dexamethasone or control, 6 h or 24 h in culture, 3 replicates	James et al., 2007
Growth plate	8	Resting, proliferating, maturing and hypertrophic zone, 2 replicates each	Isshiki et al., 2011
Primary growth plate chondrocytes	15	Control and 4 individual inhibitors,24 h in culture, 3 replicates each	Ulici et al., 2010
Growth plate	12	Resting/proliferating, maturing/hypertrophic and mineralising zone, 4 replicates each	James et al., 2010
Explant culture	18	Treatment with CNP or control, 6 days in culture, Resting/proliferating, maturing/hypertrophic and mineralising zone, 3 replicates each	Agoston et al., 2007

*The first column lists the origin of the sample. The second column indicates the amount of samples. The third column briefly summarizes the treatment and the amount of replicates. The final column indicates the reference for the samples*.

**Figure 3:**
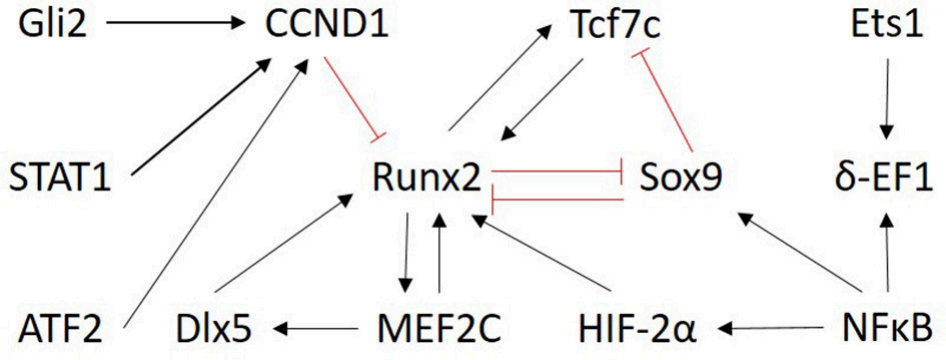
Overview of the subnetwork of factors selected for inference. The regulatory interactions are those present in the literature derived model (Kerkhofs et al., 2016). Only the nodes are considered as input for the ensemble inference. Red and black arrows indicate inhibition and activation, respectively.

Only transcription factors were chosen as input nodes for inference because their mechanism of action is more directly measured, it is less influenced by post-translational modifications and TFs are typically represented by one measurement on the microarray. No proteomic data was exploited. Selecting only the TFs from the prior network to do the inference also helped to reduce the size of the optimization problem. As a result, the network was inferred between the following 13 genes: Gli2, Tcf7, Runx2, Sox9, MEF2C, STAT1, ATF2, NFκB, CCND1, Dlx5, Ets1, δ-EF1, HIF-α2. The subnetwork from the literature-derived model that includes the components used for the inference is shown in Figure 3.

The inference methods used in the consensus approach are recapitulated in Table 2 and described in full in Supplemental Data 1. Each single method provided its own ranked list of inferred interactions. With the voting approach, a consensus network was inferred by providing a sorted list of inferred interactions according to their average rank over all the methods. To be noted also that the consensus network was built as undirected because most of the individual methods produce undirected networks. Undirected means that when a regulatory interaction is inferred, it only suggests that the interaction exists between two components but it does not presuppose which one of the components acts on the other.

**Table 2:** Summary of inference methods applied to microarray data.

**Category**	**Methods**
Statistical methods	Correlation (Pearson and Spearman), TIGRESS (Haury et al., 2012), GENIE3 (Huynh-Thu et al., 2010)
Information-theoretic methods	Mutual information, CLR (Faith et al., 2007), ARACNE (Margolin et al., 2006), MRNETB (Meyer et al., 2010)
Probabilistic methods	Bayesian (Friedman et al., 2000), GGM (Werhli et al., 2006)
Ode-based methods	Inferelator (Bonneau et al., 2006; Greenfield et al., 2013)

*The methods are divided into four categories, though the match can be somewhat arbitrary and some methods are more hybrid-like*.

#### Inference methods applied to cell fate decisions

Rapid advances in high-throughput–omics techniques over the last decade have opened a myriad of possibilities for study of biological phenomena. Inferring regulatory networks is an essential part of the general omics analyses pipeline. In Griffiths et al. (2018) the authors reviewed RNA-seq data analysis pipelines to study the developmental process in general and cell fate decisions in particular and discussed the remaining challenges such as the issue in distinguishing small biologically meaningful variations from technical artifacts. Despite this difficulty, Li et al. (2017) were able to infer a network model for growth plate chondrocytes based on 5 human patient samples. Furthermore, Li et al. (2016) succeeded in deriving a regulatory network of mouse growth plate development based on single-cell RNA-seq data. The authors developed a systematic pipeline enabling the identification of genes and signaling pathways involved in this developmental process and suggest the pipeline could be used to investigate other developmental processes.

Other inference examples include Chen et al. (2015), who proposed a tool to infer a transcriptional regulatory network from single-cell transcriptional data in order to identify operational interactions corresponding to specific cell fate determination. Importantly, the authors intended to build a Boolean network model out of it, highlighting the importance of having an “executable” model for simulation and not a simple static map (see Figure 1). Finally, Weinreb et al. (2018) developed an inference algorithm and applied it to predict cell state decision from hematopoietic progenitor cells, illustrating the range of application possibilities for regenerative medicine.

If genomic and transcriptomic data can serve for GRN inference, proteomic data might be of interest to infer protein signaling networks. Melas et al. (2014) integrated phospho-proteomic and cytokine release data to build a mechanistic model of signal transduction in the adult chondrocyte. They inferred regulatory protein interactions directly from the data in order to identify previously reported as well as new key players involved in chondrocyte homeostasis. This could be very beneficial to find important biological factors with a potential for cartilage regeneration.

## Combining knowledge-derived model with network inference: case study applied to chondrocyte differentiation

### General principles

It should be kept in mind that both knowledge-driven and data-driven approaches can be relevant depending on the type of questions the model is supposed to answer and should be chosen wisely in a problem-centric approach (Janes and Lauffenburger, 2006). An alternative can be to mix both of them as was done for instance in Melas et al. (2011) where the authors developed a curated knowledge-derived topology linking external stimuli to signaling pathways, and subsequently used data-driven approaches to link signaling pathways to cellular responses. Following this approach, they produced an extended network that was shown to perform better in predicting qualitative cellular responses to specific cues than a purely data-derived model.

**Figure 4:**
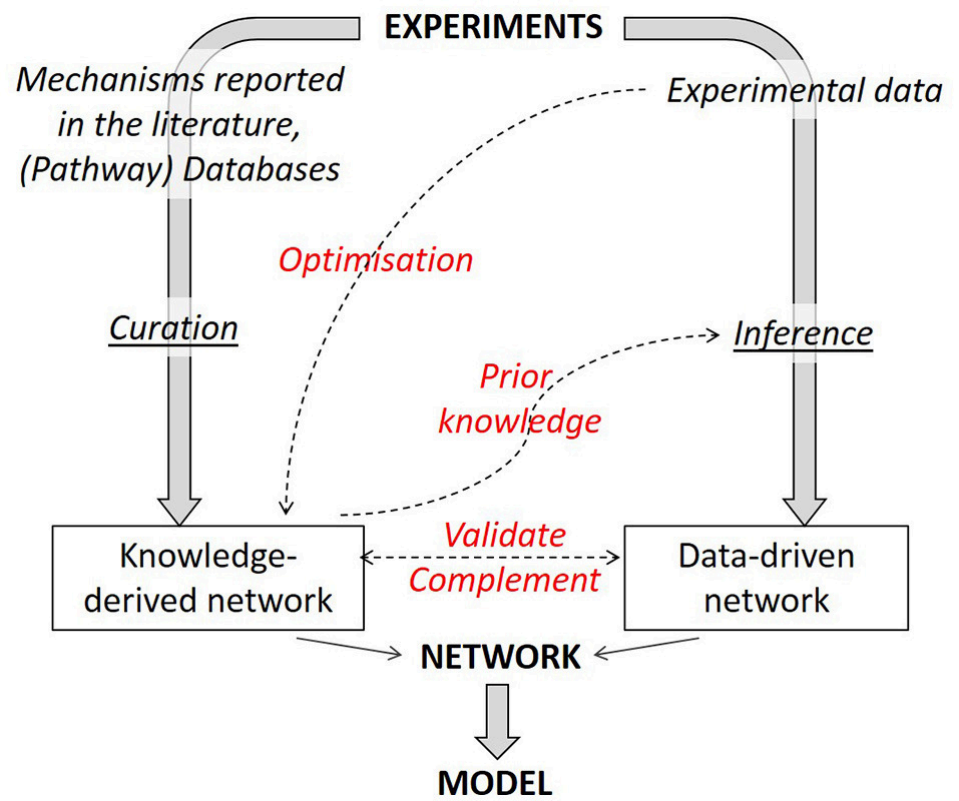
Strategies to combine knowledge-derived and data-based modeling. When deriving a network graph from experimental results, different routes can be followed, either a mechanistic one (using available knowledge) or a data-driven one. Combining both strategies allows to obtain an optimized network graph. Knowledge-derived networks can be fitted against experimental data to optimize network parameters. Knowledge-derived networks can serve as prior knowledge within some inference algorithms in order to improve inference performance. Finally, data-driven networks can be used to validate or complement (add or remove interactions) a knowledge-derived one and vice versa.

It is not only possible to link together literature-derived and data-based networks but one method can benefit from the insights gained with the other (Figure 4). There are two reciprocal benefits: (1) literature-derived models can gain from data-based optimization and (2) network inference algorithms can see their performance considerably increased by incorporating prior knowledge in the inference process.

With respect to the first benefit, one should bear in mind that molecular regulatory network models not only serve as a descriptor, but also as a predictor of the cell response. One of the limits of logical models is their lack of accuracy in yielding useful predictions and new insights, In Saez-Rodriguez et al. (2009), the authors explain how to turn network models into predictive logical models that are rigorously calibrated against experimental data. By training a general literature-derived network against cell specific data, they succeeded in identifying interactions that did not seem to be functional in the specific cell type under study. Additionally, they could propose new interactions not present in the initial generic model although being supported by other sources from literature.

As for the second benefit, there are indeed techniques that aim at inferring regulatory network from experimental data whilst incorporating curated knowledge as a prior within the algorithm in order to increase performance. For instance, for the constructions of a predictive model of signal transduction in chondrocytes, Melas et al. (2014) combined the proteomic data with a priori knowledge of the proteins' connectivity. This is also the case in the “Inferelator” algorithm (Bonneau et al., 2006) which infers transcription factor—gene interactions.

Having the most reliable network possible is important since the dynamic of the resulting model will settle down into equilibrium states strictly complying with the topology of the regulatory interactions. Hence, the topology will define the outcome of the simulation. To illustrate the interactions as depicted in Figure 4 (compare, validate, optimize, prior knowledge), we will return one more time to our growth plate example discussed in the previous sections.

### Case study: chondrocyte differentiation in the growth plate

#### Comparing different networks

In a direct approach, the interactions with the highest combined rank in the consensus-inferred network can be compared to the interactions present in the knowledge-derived network. By doing so, we found most of the interactions present in both networks (Kerkhofs, 2015), which can be interpreted as a corroboration of the knowledge-derived network. Additionally, we noticed that some interactions inferred with a high rank were not present in the literature-derived topology (for instance MEF2C-HIF-2a). This can be considered as a suggestion for complementing the knowledge-derived network.

Besides this direct comparison as mentioned in the previous paragraph, classical measures of performance can be used to assess rigorously the match between the micro-array data inferred network (discussed in section Data Driven Modeling—Network Inference) and the literature-derived network (discussed section *in silico* Knowledge-Based Modeling of Regulatory Networks to Study Cell Fate Decision). These measures include the receiver operating characteristic (ROC) curve, the Precision Recall (PR) curve and their respective area under the curve (AUROC and AUPR), see Supplemental Data 1 for detailed definitions. They are based on the comparison between an inference algorithm and a gold standard and they are commonly used in the context of GRN inference (Marbach et al., 2012). In the chondrocyte network study (Kerkhofs, 2015), the main objective was to assess to what extent the topology inferred from micro-array data corresponds to the knowledge-based topology manually curated from the literature. Practically, the inferred network and the literature-derived network were compared while regarding the literature-derived network as a pseudo-gold standard.

In parallel, the inferred network was also compared to another network topology derived from an online database named STRING (Search Tool for the Retrieval of Interacting Genes/Proteins). The STRING network is not the result of a mathematical inference, instead, the STRING tool generates networks for which the connections represent functional associations (both direct and indirect) predicted from screening large databases containing protein-protein interactions gathered from genomic context predictions, text-mining from PubMed and co-expression data. The STRING network presented here was generated by querying the STRING tool, giving as an input the 13 proteins and the total number of interactions of the network depicted in Figure 3. Each interaction included in the obtained STRING network is annotated with a bibliographic or experimental reference to the study that enabled adding the interaction to the network. For a detailed description about the tool, we refer the readers to Szklarczyk et al. (2017). The aforementioned inferred network was tested against the STRING topology to assess whether the inferred network was better at fitting the literature-based model than at fitting the automatically produced STRING topology. In other words, it was tested to assess whether the manual literature curation and topology creation was superior to an automatic one in the cell specific context of a growth plate chondrocyte.

**Figure 5:**
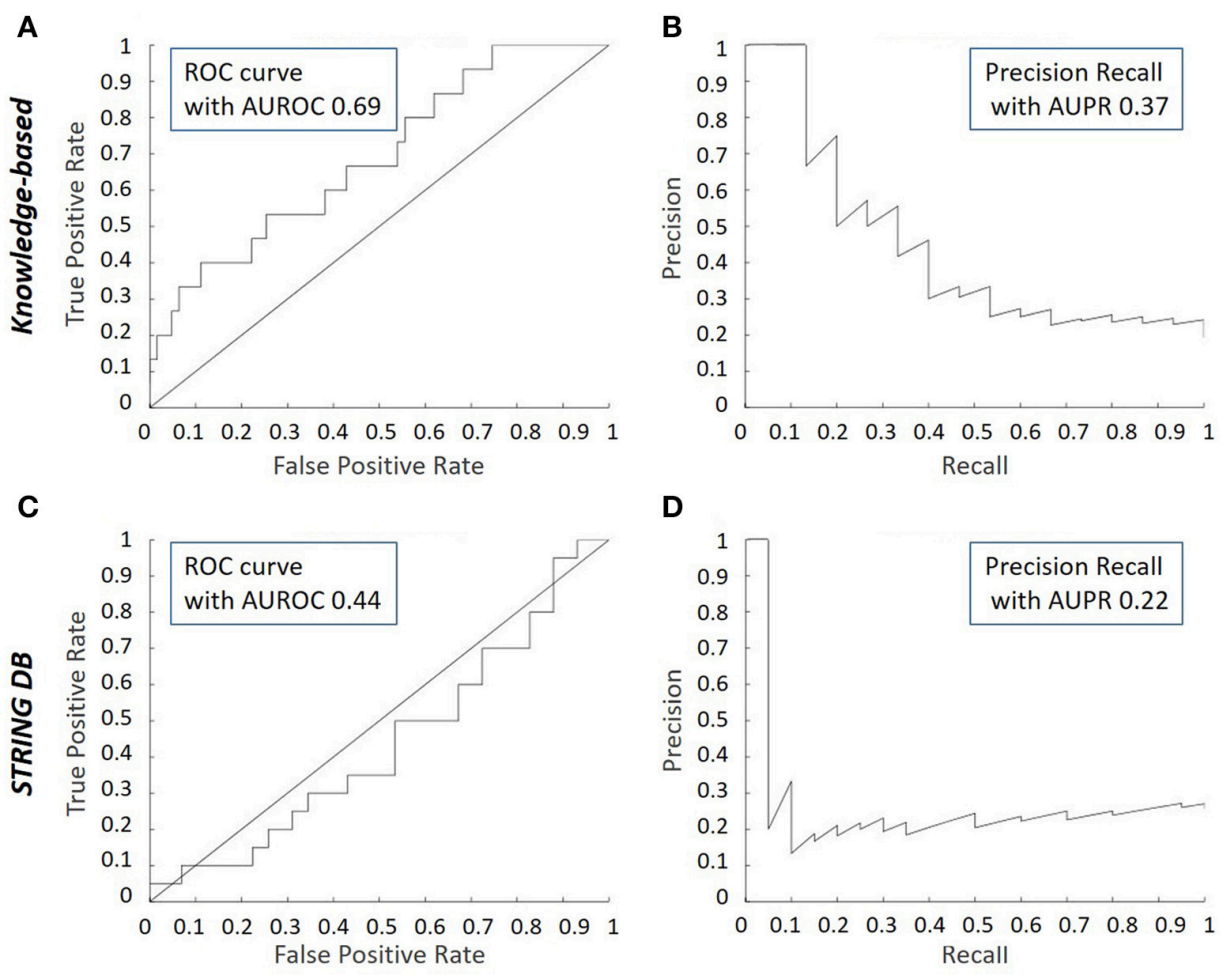
Receiver Operating Characteristic (ROC) and Precision Recall (PR) curves for inferred consensus network with respect to knowledge-based network and STRING network. The ROC plots the True Prediction Rate (TPR) against the False Prediction Rate (FPR) for each (cumulative) interaction inferred. The PR curve plots the precision vs. the recall for each (cumulative) interaction inferred. **(A)** ROC curve for the inferred consensus network compared to the literature-derived topology. **(B)** PR curve for the inferred consensus network compared to literature-derived topology. **(C)** ROC curve for the inferred consensus topology compared to the STRING network. **(D)** PR curve for the inferred consensus topology compared to the STRING network.

When comparing the ROC curves of the inferred network with respect to either the knowledge-based or the STRING derived model, it can be appreciated that the performance is better with the knowledge-based network (Figures 5A,C). Indeed, the concavity of the curve points toward a good correspondence between the inferred interactions and the ones in the knowledge-based network, whereas a line with a 45° slope through the origin, as is the case when comparing with the STRING topology, indicates a random performance. This is quantitatively confirmed by the higher AUROC value of the inferred vs. the knowledge-based network curve (0.69) compared to the inferred vs. STRING network curve (0.44). It means the literature-derived network depicted in Figure 2 was able to explain correctly an important part of the experimental behavior at the transcriptomic level. The superior behavior of the literature-derived network over the STRING network could be due to the latter including indirect interactions whereas the former mostly sticks to direct interactions. Another significant difference between the two networks was that most of the interactions included in the knowledge-derived network were derived from experiments on growth plate chondrocytes or a closely related cell type (Kerkhofs and Geris, 2015). In contrast, interactions from a wide variety of contexts, often from cancer cells, served to build the STRING database and the subsequent network. When comparing the precision-recall curves and the AUPR values (Figures 5B,D), it was clear that the knowledge-based network again outperformed the STRING network at least for the first few guesses (perfect PR being a straight line passing through the upper right corner with AUPR of 1). Overall, this section illustrates the fact that tools which automatically generate networks from public database curation may serve as a good starting point for regulatory network construction but also that this strategy is still missing the cell type and biological context specificity. This is particularly important for biological processes that are studied by smaller communities and for which less context-specific information is available in the public databases (which is the case for growth plate biology when compared with cancer biology). Indeed, automatic database curation with software tools may offer a systematic and more objective way to build networks from published information but it is very likely much improved by “manual” construction/adaptation of the network for specific biological questions.

#### Chondrocyte network inference with integration of prior network

A number of inference algorithms permit the inclusion of known interactions as input to the inference process. The input knowledge usually takes the form of a matrix containing as many lines and columns as the number of factors, where the value at each location in the matrix indicates the presence/absence, direction and strength of the interaction between the factors depicted in that particular row and column. For a detailed explanation on the implementation of prior knowledge, see Supplemental Data 1. This use of prior knowledge will increase the probability of finding models that have a bigger similarity with the prior network. Of course, to keep the benefit of the data-inference from experimental data, a balance must be struck between forcing compliance with the prior network and fitting the data.

For the chondrocyte differentiation network the incorporation of prior knowledge was investigated through the sole use of the Inferelator algorithm as described in Greenfield et al. (2013). This method tunes influence of prior knowledge on the outcome through an adjustable parameter *g*. Since part of the literature-derived network was used as prior knowledge, the effect of the parameter *g* on the correspondence between the inferred and the literature-derived network (AUROC) was investigated, together with the error between experimental data and simulated values (SSR, see Supplemental Data 2). It was a way to identify which *g*-value was producing a model that matched the prior information without sacrificing compatibility with the microarray data.

In Kerkhofs (2015), we did a screening of different values for the parameter *g* in order to select a value so that the adherence to the prior network was not too strict. Therefore, the algorithm could infer new interactions explaining the experimental data while taking into account the prior knowledge about chondrocyte biology in the growth plate. Table 3 reports the 11 regulatory interactions with the highest incidence in the inference process. Similarly to the consensus inference (section Inference of a Growth Plate Network Following a Consensus Approach), the integration of prior knowledge identified some high-ranking inferred interactions that were absent from the prior knowledge-based network such as the activation of Tcf by MEF2C (MEF2C → Tcf7), Tcf7 → HIF-2α, and HIF-2α → δ-EF1. Some of these predicted interactions were in fact already described in literature such as MEF2C forming an enhanceosome with Tcf7 and other factors to upregulate the Runx2 activity in a synergistic way (Kawane et al., 2014). Therefore, the network may benefit from being supplemented with these interactions. However, for other predicted interactions, only weak evidence was present in the literature. Hence, the inference may provide an indication for subsequent experimental investigation into the veracity of those predicted interactions. Highlighting such interesting new interactions may make the model more accurate with respect to the data and possibly reinforces its predictability. Finally, the knowledge-based topology might also be improved through the exclusion of regulatory interactions that do not score well in the data-based analysis.

**Table 3:** Inferred interactions with inferelator and prior knowledge (*g* = 5).

**Source**	**Target**	**StoT**	**TtoS**	**Pearson**	**StoTorig**	**TtoSorig**
MEF2C	Runx2	0.97	0.91	0.69	1	1
δ-EF1	Ets1	0.16	0.88	−0.38	0	1
CCND1	Atf2	0.02	0.91	0.61	0	1
Sox9	Runx2	0	0.90	−0.30	−1	−1
Dlx5	MEF2C	0.02	0.84	0.32	0	1
MEF2C	Tcf7	0.77	0	0.59	0	0
HIF-2α	Tcf7	0.14	0.51	0.62	0	0
HIF-2α	δ-EF1	0.56	0	0.39	0	0
NF-κB	Sox9	0.55	0	0.40	1	0
HIF-2α	NF-κB	0	0.53	0.04	0	1
HIF-2α	MEF2C	0	0.44	0.54	0	0

*Selection of the first ranked interactions. StoT is the fraction of times where a directed interaction from the source (1st column) to target (2nd column) is found in the bootstrap procedure. TtoS is the fraction of cases where a reverse directed interaction is found. The fifth column gives the Pearson correlation in the microarray dataset. StoTorig is the directed interaction from the source to target in the literature-derived network (Figure 2). TtoSorig is the reverse interaction. 0 indicates no interaction, 1 is an activation and −1 is an inhibition*.

This illustration of network inference on the one hand shows how data-driven approaches may automatically, and relatively quickly, exploit experimental datasets to build interconnected network of gene and protein interactions. However, using these networks to propose further experiments is not straightforward since the output remains a static map. On the other hand, manual curation of networks created through a bottom-up approach is a very fastidious and time-consuming process, but these networks easily allow dynamic analyses and simulations that suggest new wet lab experiments. Hence, there is a necessity to develop methods that allow combining both approaches into a single framework having the best of both worlds as suggested by Poirel et al. (2013).

In the context of osteochondral regenerative medicine, the ultimate goal of reconstructing such network of regulation is to obtain a predictive model in order to gain understanding into the signaling or regulatory mechanism controlling cellular behavior and/or physiology of degenerative diseases. It might enable the identification of key factors for therapeutic targeting ensuring reliable cellular differentiation for tissue engineering applications or to propose potential disease-modifying therapies in the context of cartilage degenerative disease such as osteoarthritis (Hopkins, 2008). Application of such a methodology to the growth plate system already suggested new interactions and some factors as key regulators in the phenotypical transition of chondrocytes. For instance, in the inference previously presented (Kerkhofs, 2015), one of the highly ranked inferred interactions that was not present in the prior network, was NFkB-MEF2C with a negative correlation. MEF2C knockdown was shown to increase NFkB activity in endothelial cells (Xu et al., 2015) but no mRNA measurement was performed. This could constitute a suggestion of experimental design for validation of the *in silico* result. Finally, dynamic simulations associated with this growth plate network showed that *in silico* knock-out of NFkB was decreasing the reachability of the hypertrophic state in the optimized model, i.e., making it less likely for a cell to be in that state. The factors that were absolutely required to reach the hypertrophic state, as in their absence no Runx2+ stable state was found, were Ras, Ihh, Gli2, and FGF. The activation of Smad7 in the model was the most expedient way to remove the Sox9+ (proliferative) phenotype. Hence, according to the model, inhibiting Smad7 could be a strategy to decrease initiation of hypertrophic differentiation; or activation of Ras, Gli2, or FGF pathways might help to promote hypertrophy in order to produce sustainable bone TE constructs.

## Conclusion

As new computational tools become available, an increasing number of biological systems is investigated computationally with increasing effectiveness. Computational approaches have already shown their relevance in the field of regenerative medicine with models of bioreactor studies and biomaterial design. However, this relevance also holds for the models focusing on the intracellular level, through the use of GRN models to understand the cellular decision-making processes, e.g., in the context of cell differentiation. Understanding and controlling these processes is a necessary step to increase quality and robustness of TE constructs. For the specific case of osteochondral tissue engineering, only few cell signaling or signal-response models have been reported in the literature up to date. We have reviewed our own published work on computer modeling of differentiation of growth plate chondrocytes to illustrate the potential of *in silico* approaches in designing proper culture strategies to control the transition of these chondrocytes from a proliferative to a hypertrophic phenotype and find those (potentially druggable) targets. Such targets would allow preventing a switch from the proliferative to the hypertrophic phenotype in the case of cartilage degenerative diseases such as osteoarthritis. Correcting aberrant cellular behavior with drugs requires knowledge about multiple interacting signaling proteins, which necessitates the use of computer tools (Kumar et al., 2006; Hopkins, 2008; Voit, 2012).

The last section of this paper illustrate the benefits that network inference from experimental data could bring to predictive computational models. The main obstacle currently preventing the expansion of *in silico* approaches is the lack of informative and quantitative experimental data. Indeed, modelers face the difficulty of obtaining data with a sufficiently high information content, such as perturbation data from human samples, to build and validate their models.

*In silico* modeling does not aim to replace traditional experimental methods but provides an additional tool to interpret the results obtained in those experiments and to suggest new informative experiments, as an integral part of the experimental research cycle. The objective of the models described in this review is to bring a higher level of understanding, increase time efficiency of experimentation and decrease costs in the process of therapy development.

## Author contributions

JK and LG conceived and designed the models. RL and JK performed the simulations. RL, JK, and LG analyzed the results and wrote the paper.

### Conflict of interest statement

The authors declare that the research was conducted in the absence of any commercial or financial relationships that could be construed as a potential conflict of interest.
