# Supplementary material for: Computational Modeling and Reverse Engineering to Reveal Dominant Regulatory Interactions Controlling Osteochondral Differentiation: Potential for Regenerative Medicine
Source: Front Bioeng Biotechnol. 2018 Nov 13;6:165. doi: 10.3389/fbioe.2018.00165 (PMC6243751; doi:10.3389/fbioe.2018.00165)
Supplement: Supplementary file 1 [file Data_Sheet_1.PDF]

frontiers

Supplementary Material: Computational modeling and reverse engineering to reveal dominant regulatory interactions controlling osteochondral differentiation: potential for regenerative medicine

Supplemental data 1: Description of inference methods

This section introduces briefly the general principles of each inference method associated with few exemples of published algorithms which belong to these categories. More details about the mathematical framework of each method can be found in the following section.

The statistical methods gather the correlation methods and the regression methods. The former aims to assess some correlation coefficients such as Pearson's or Spearman's in order to rank the edges of the network. The second tries to identify transcription factors as regulators according to the target gene's expression with a sparse linear regression followed by resampling. For instance, TIGRESS (Trustful Inference of Gene REgulation using Stability Selection)(Haury et al., 2012), LARS (Last Angle Regression) and GENIE3 (Huynh-Thu et al., 2010) are regression-based algorithms. See supplemental material S1 for a detailed description.

Probabilistic methods include the graphical Gaussian Model (GGM) and the Bayesian inference. This last one uses Bayes' theorem to optimize the probabilities of possible networks using heuristic searches (Box. 1) and by assessing dependencies between random variables corresponding to the nodes of the network. Therefore, it optimizes (maximizes) the likelihood that the network, given in output, produces the data set.

The mutual information or information theoretic methods rank edges based on how much Mutual Information they contain and then filter them for causal relationships. This is the case for some off-the-shelf inference methods such as CLR (Contexte Likehood of Relatedness) (Faith et al., 2007) or ARACNE (Algorithm for the Reconstruction of Accurate Cellular Networks) (Margolin et al., 2006). Typically, the nodes are linked in the network if information of one variable is influenced by the knowledge of the value of the others. It is based on the information theory in which the Mutual Information (MI) is a measure of the amount of information obtained about one variable, through another variable.

ODE based-approaches tempt to predict the change values of the expression based on current expression. These methods try to infer qualitative information and dynamics from time series data. See supplemental material S1 for a detailed description.

1 RELEVANCE NETWORKS

A basic strategy is to analyse relations between the nodes (node and gene are used interchangeably for members of the network) one pair at a time. Any measure of distance or similarity can be used to compute association scores. To infer a final network, a threshold is chosen, with the aim of excluding spurious and indirect associations. In the implementation of Butte and Kohane (2003), mutual information and correlation are suggested as suitable scores. The construction of a relevance network is computationally

cheap, but these methods do not take into account the context of the network (Werhli et al., 2006). We have ranked the edges according to Pearson correlation, Spearman rank correlation and mutual information. Pearson correlation is calculated according to:

$$r_{ij} = \frac{\sum_{k=1}^{M}(x_{ik} - \overline{x_i})(x_{jk} - \overline{x_j})}{\sqrt{\sum_{k=1}^{M}(x_{ik} - \overline{x_i})^2 \sum k = 1^M (x_{jk} - \overline{x}_j)^2}}$$

where M is the number of samples, and i and j are nodes and x is the average expression value over all samples. For the Spearman rank correlation, expression values for a particular node (x) are replaced by their rank. The Pearson correlation measures linear relationships between variables, the Spearman correlation is more suited for (monotonic) nonlinear effects. Mutual information is given by:

$$I(X_i, Xj) = \int\int p(x_i, x_j) \log \frac{p(x_i, x_j)}{p(x_i)p(x_j)}\, dx_i\, dy_j$$

This is a measure of how much information is shared between two variables. if two variables are independent, then p (xi, xj) = p (xi) p (xj) and the mutual information is zero. If knowing xi entails a better estimate of xj , the value will be higher than zero. To calculate this integral for a dataset, expression values have to be binned (discretized to a number of contiguous bins). However, the estimate of the integral obtained through this process is biased as, in calculating the logarithm, errors due to an underestimation have a higher effect than those due to overestimation. Sales and Romualdi (2011) tested estimators on non-linear data and found the method of Kraskov et al. (2004) had the best performance. This algorithm estimates mutual information form the distance to the k nearest neighbours. Mutual information is estimated by:

$$I(X_i, Xj) = \psi(k) - \frac{1}{k} + \overline{\psi(n_{x_i}) + \psi(n_{x_j})} + \psi(M)$$

where n_x is the number of points falling in radius ϵ (the distance of the kth nearest neighbour) in subspace X and the digamma function $\psi(x) = \frac{\Gamma'(x)}{\Gamma(x)}$, which satisfies $\psi(x+1) = \psi(x) + \frac{1}{x}$. For each data point, ϵ and the number of points n_{x_i} and n_{x_j} are determined. To obtain the mutual information estimate, the result is averaged over all data points. This method was used with k=3 (shown to have good performance in Sales and Romualdi (2011)). These measures assume that samples are independent, and in principle can only deal with time series if time between samples is sufficiently long.

2 INFORMATION-THEORETIC METHODS

The information-theoretic methods used in this work utilize mutual information as a starting point to build the inferred network. We discuss them each in turn.

2.1 CLR

Context likelihood of relatedness, or CLR, derives a score from the mutual information matrix for each interaction by determining how unusual a certain value is for the particular genes (Faith et al., 2007). The score hence assesses the value against the background values for the gene. CLR computes the score S

$$S = \sqrt{Z_i^2 + Z_j^2}$$

for a pair of nodes i and j. Z represent z-scores derived from the mutual information matrix as:

$$Z_{ij} = max\left(0; \frac{I_{ij} - \overline{I_i}}{\frac{1}{N}\sum_j^N \sqrt{(I_{ij} - \overline{I_i})^2}}\right) \tag{S1}$$

Where I_{ij} are entries in the mutual information matrix (i.e. the mutual information of node i and node j) and $\overline{I_i}$ is the average mutual information value for node i. Note that CLR exploits the sparsity of the inverse problem that justifies the assumption that most genes do not regulate each other, hence the collection of all mutual information values of a particular gene gives an idea of background mutual information values. This estimate may be more inaccurate using only a limited number of genes, but CLR was shown to perform well on a network of 10 nodes (Lingeman and Shasha, 2012).

2.2 ARACNE

ARACNE, short for Algorithm for the Reconstruction of Accurate Cellular Networks, relies on the data processing inequality to break up indirect interactions in node triplets. The data processing inequality states that if a gene X_i and X_j interact (exclusively) through a third gene X_k, then:

$$I(X_i, X_k) \leq min(I(X_i, Xj); I(Xj, Xk))$$

Thus in this case the lowest mutual information value comes from an indirect interaction. To prune indirect interactions, the interaction with the lowest mutual information value of is removed for all triplets in the network. Each triplet is examined simultaneously, so the order of removing reactions has no effect on the results (Margolin et al., 2006). Note that the data processing inequality can also be satisfied when X_i and X_j interact directly (Bansal et al., 2007). Hence, true interactions might be discarded. To lower the likelihood of this occurrence, no interactions are discarded if the lowest value lies within 15% of the second lowest. The original version of ARACNE calculates mutual information via a kernel density-based method, here the approach of Kraskov et al. (2004) is used.

2.3 MRNETB

MRNETB (Minimum Redundancy NETworks Backward) relies on the maximum relevance minimal redundancy (MRMR) criterion. For each target node, the set of predictors with maximal pairwise relevance with the target gene is sought. At the same time, these predictors must have a high pairwise independence (Meyer et al., 2010). Relevance u and redundancy r are determined by the mutual information values:

$$u = \frac{1}{|S_i|} \sum_{j \in S_i} I(X_i, X_j)$$

$$r = \frac{2}{|S_i|(|S_i| - 1)} \sum_{j,k>j \in S_i} I(X_j, X_k)$$

where S_i is the selection of predictors for target node i. Initially, all predictors belong to the selection. At each step, the algorithm removes the predictor whose removal begets the lowest value of the objective function $(u - r)$. After each such step, an iterative sequential replacement procedure substitutes a selected predictor with a non-selected one so that the objective function is optimally increased. This procedure stops

when no further improvement by replacement is possible. The algorithm converges when the relevance term becomes higher than the redundancy term.
