# Supplementary material for: Computational Modeling and Reverse Engineering to Reveal Dominant Regulatory Interactions Controlling Osteochondral Differentiation: Potential for Regenerative Medicine
Source: Front Bioeng Biotechnol. 2018 Nov 13;6:165. doi: 10.3389/fbioe.2018.00165 (PMC6243751; doi:10.3389/fbioe.2018.00165)
Supplement: Supplementary file 2 [file Data_Sheet_2.PDF]

Supplementary Material

Computational modeling and reverse engineering to reveal dominant regulatory interactions controlling osteochondral differentiation: potential for regenerative medicine

R.Lesage*, J.Kerkhofs, L.Geris*

*** Correspondence:** Corresponding Author: liesbet.geris@uliege.be

1 Supplementary Data n°2: Measures of performance for inferred networks.

Modified from (Kerkhofs, 2015).

In the field of machine learning, a set of standard tools exists in order to assess a given inference algorithm performance. In such a case, the inferred network is typically compared to some gold standard. However, in the context of the presented case study, we do not intend to test inference algorithms' performance but to use some of those algorithms to test the estimated literature derived network against micro-array data. Indeed, we consider that the consensus network, inferred with algorithms, correctly mirrors the information inherent to the micro-array data. Given the aforementioned, the literature-derived network is considered to be the 'estimated' network and the inferred network is considered to be the (pseudo) gold standard network in the following definitions.

A true positive (TP) edge is an edge that is correctly estimated in the literature-derived network when comparing with the inferred network. A false positive edge (FP) is an estimated edge that is absent in the gold standard. Similarly, a true negative (TN) edge is correctly predicted to be absent in the network. A false negative (FN) edge is present in the gold standard, but is not present in the estimated network. Precision (P) is given as:

$$Precision = \frac{TP}{TP + FP}$$

Precision indicates what percentage of estimated edges are correct. Recall (R) is defined as:

$$Recall = \frac{TP}{TP + FN}$$

Hence, recall is the percentage of interactions in the gold standard that have been estimated according to litterature. Recall is also known as the true positive rate (TPR). The false positive rate (FPR) is:

$$False\ Positive\ Rate = \frac{FP}{FP + TN}$$

which is the percentage of edges, not present in the gold standard, that have been estimated to be present. When a sorted list of edge prediction is available in conjunction with a golden standard, the performance can be visualised by a receiver operating characteristic (ROC) curve or by a precision-recall (PR) curve. Specifically, the inclusion of the first x edges is used to plot the xth point of the curve. The ROC curve plots the TPR against the FPR at each point x. As such, it portrays how much of the gold standard network correctly estimated versus how much 'wrong' edges have been included. The more concave this curve is, the better the estimation from litterature (Lingeman and Shasha, 2012) and the better the literature derived network reflects the micro-array data. With random guesses, this percentage will be approximately equal and consequently a random performance corresponds to line with a 45 slope through the origin. A PR curve plots the precision versus the recall, i.e. how many guesses are correct versus the percentage of 'correct' edges included. The longer the precision stays close to 1, the better the performance is. A random performance equates to a horizontal line at a level corresponding to the chance of a random guess being correct $\left(\frac{TP+FN}{TN+FP}\right)$. The PR curve lays more emphasis on the initial precision of results, (i.e. whether the first guesses are correct) whereas the ROC curve focuses on the entirety of results (Haury et al., 2012). From these curves, a single measure of performance can be derived, namely the area under the PR curve (AUPR) and the area under the ROC curve (AUROC). An AUROC of 0,5 corresponds to random guesses.

Box : Definitions related to comparison of networks In the field of machine learning, a set of standard tools exists in order to assess a given inference algorithm performance. An inferred network is typically compared to some gold standard. In the context of the presented case study, the literature-derived network is considered to be the (pseudo) gold standard network in the following definitions.

True Positive Rate (TPR) or sensitivity or recall: fraction of edges present in the gold standard network that are also present in the inferred network.

False Positive Rate (FPR): fraction of edges absent in the gold standard network that are present in the inferred network.

Precision: fraction of edges present in the inferred network that are also present in the gold standard (fraction of correctly predicted edges)

Receiver-Operator Curve (ROC): The ROC curve plots the TPR against the FPR for an increasing number of edges present in both networks.

Precision-Recall (PR): The PR curve plots the Precision against the Recall (or TPR) for an increasing number of edges present in both networks.

AUROC and **AUPR** are single values of performance that can be derived from the ROC and PR curves respectively by calculating the area under (AU) the curves.

Bootstrapping: A resampling strategy. When doing an analysis with a data set, it is common to calculate measures of dispersion such as variance, standard errors and confidence intervals. The bootstrapping serves to evaluate the sensitivity of these measures to the specificity of the sample used, by analyzing the possible sub-samples. For instance, it serves to avoid over-fitting and

sampling errors since the analysis will be done several times with different sub-part of the available sample. It is particularly convenient when the data set used to train an algorithm is not big enough.

The **sum of squared residuals (SSR)** is a measure of error between measured values and predicted/simulated values. In the case of the inferelator, once it has inferred a ranked list of edges, the algorithm translates the interactions in a set of ODEs to simulate the system's evolution. Hence, it produces simulated values of the entities' activity, which can be compared to the measured values in order to estimate the error. Here, the inferelator enables the calculation of an SSR between the data and simulated values. Thanks to a bootstrapping procedure, several SSR values are calculated, each for a different sub-sample of the data set. The SSR is therefore represented by its mean and the standard deviation over the sub-samples.
